# Brain Shift in Neuronavigation of Brain Tumors: An Updated Review of Intra-Operative Ultrasound Applications

**DOI:** 10.3389/fonc.2020.618837

**Published:** 2021-02-08

**Authors:** Ian J. Gerard, Marta Kersten-Oertel, Jeffery A. Hall, Denis Sirhan, D. Louis Collins

**Affiliations:** ^1^Department of Radiation Oncology, McGill University Health Centre, Montreal, QC, Canada; ^2^Department of Computer Science, Concordia University, Montreal, QC, Canada; ^3^Department of Neurology and Neurosurgery, McGill University, Montreal, QC, Canada

**Keywords:** brain shift, neuronavigation, intra-operative ultrasound, registration, neurosurgery, image-guided neurosurgery

## Abstract

Neuronavigation using pre-operative imaging data for neurosurgical guidance is a ubiquitous tool for the planning and resection of oncologic brain disease. These systems are rendered unreliable when brain shift invalidates the patient-image registration. Our previous review in 2015, *Brain shift in neuronavigation of brain tumours: A review* offered a new taxonomy, classification system, and a historical perspective on the causes, measurement, and pre- and intra-operative compensation of this phenomenon. Here we present an updated review using the same taxonomy and framework, focused on the developments of intra-operative ultrasound-based brain shift research from 2015 to the present (2020). The review was performed using PubMed to identify articles since 2015 with the specific words and phrases: *“Brain shift” AND “Ultrasound”*. Since 2015, the rate of publication of intra-operative ultrasound based articles in the context of brain shift has increased from 2–3 per year to 8–10 per year. This efficient and low-cost technology and increasing comfort among clinicians and researchers have allowed unique avenues of development. Since 2015, there has been a trend towards more mathematical advancements in the field which is often validated on publicly available datasets from early intra-operative ultrasound research, and may not give a just representation to the intra-operative imaging landscape in modern image-guided neurosurgery. Focus on vessel-based registration and virtual and augmented reality paradigms have seen traction, offering new perspectives to overcome some of the different pitfalls of ultrasound based technologies. Unfortunately, clinical adaptation and evaluation has not seen as significant of a publication boost. Brain shift continues to be a highly prevalent pitfall in maintaining accuracy throughout oncologic neurosurgical intervention and continues to be an area of active research. Intra-operative ultrasound continues to show promise as an effective, efficient, and low-cost solution for intra-operative accuracy management. A major drawback of the current research landscape is that mathematical tool validation based on retrospective data outpaces prospective clinical evaluations decreasing the strength of the evidence. The need for newer and more publicly available clinical datasets will be instrumental in more reliable validation of these methods that reflect the modern intra-operative imaging in these procedures.

## Introduction

**Figure 1:**
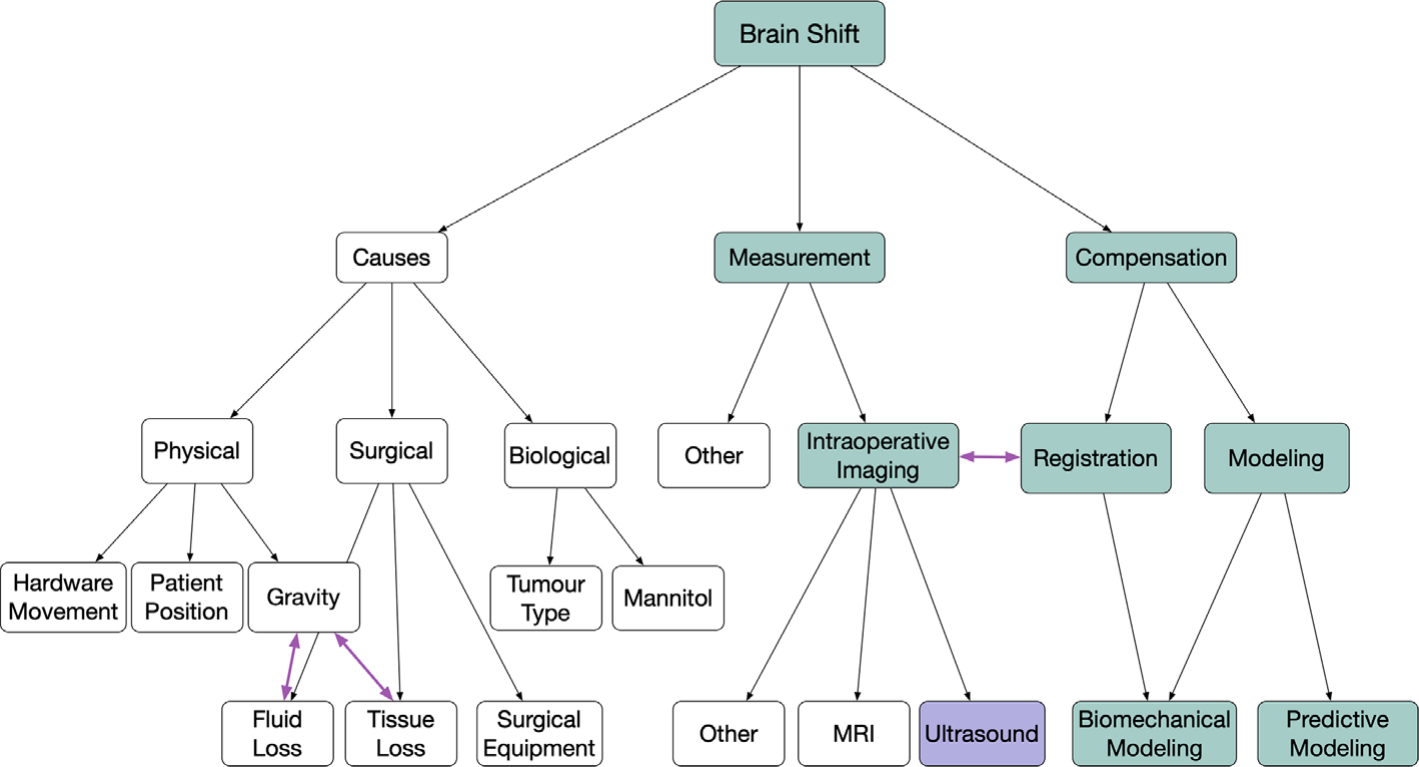
Highlighted flow chart following classification from (1) showing the focused coverage of this review.

Neuronavigation using pre-operative imaging data for neurosurgical guidance is a ubiquitous tool for the planning and resection of oncologic disease in the brain and has become common practice in many centers. It is well known that these systems are rendered unreliable when brain shift is present. Any factor, physical, surgical, or biological, that violates the rigid body assumption of neuronavigation causes the tissues of the brain to shift and move away from the pre-operative images creating a difference between the reported location of anatomy in the virtual image and patient spaces. Simply put, brain shift invalidates the patient-to-image mapping (1). In our previous 2015 review of brain shift in neuronavigation (1), we offered a new taxonomy, classification system, and a historical perspective related to the causes, measurement, and pre- and intra-operative compensation of this phenomenon. In this work, we present an updated and focused review using the same taxonomy and framework on the developments of intra-operative ultrasound-based brain shift applications over the last five years, *i.e.* from 2015 to the present. A visual representation of the previously described classification system along with the highlighted trajectory of the focus of this review can be seen in **Figure 1**.

The first use of A-mode (1D) ultrasound (US) for adult neurosurgery was completed by Dr. William Peyton in 1951 and reported by Wild and Reid in 1953 (2). The first use of B-mode (2D) US in adult neurosurgery of the spine was in 1978 by Reid (3) and in the brain in 1980 by Rubin et al. (4). In the latter, they observed intra-cranial anatomy with real-time ultrasound as well as a grade III astrocytoma and postulated that there may be benefit for this technology as a tool for surgical planning and biopsy procedures. Since then, and throughout the 2000s, intra-operative ultrasound (iUS) has been used in many capacities to evaluate, quantify, and correct for brain shift and modify surgical plans in real-time without the use of ionizing radiation exposure (*e.g.* from CT) all while minimizing any disruption to the surgical workflow. Over the last 5 years the rate of publication for intra-operative based ultrasound intervention for brain shift evaluation, quantification, and correction has dramatically increased. In the context of these advances, we review the current state, potential, and challenges that remain in the context of iUS for neuronavigation of brain tumors.

## Brain Shift Taxonomy

In order to assist with the clarity of the review and the discussions to follow, this review follows the same taxonomy and classification system as the 2015 publication: *Brain shift in neuronavigation of brain tumours: A review* (1). To begin, brain shift is defined as—*any factor, physical, surgical, or biological, that violates the rigid body assumption of neuronavigation creating a difference between the reported location of anatomy in the virtual image and patient spaces*. The discussion of brain shift is further separated into three categories; 1) factors that cause brain shift, 2) methods for quantifying brain shift, and 3) methods to correct or account for brain shift, followed by more specific subclassifications. As highlighted in **Figure 1**, the articles in this review are primarily those that describe either the measurement or compensation of brain shift using intra-operative ultrasound imaging in the context of image registration, biomechanical modeling, or predictive modeling.

## Intra-Operative Ultrasound for Neurosurgery

Ultrasound imaging uses high frequency sound waves that are emitted and detected by different probes and transducers. In the context of neurosurgery, the optimal choice of transducer and type of acquisition frequency will depend on the location and sonographic properties of the lesion of interest, the size of the craniotomy in which the probe can be placed, the surrounding anatomy, and of course, surgeon preference. The intensity of structures in these images directly reflects the amplitude of the detected signal driven by micro reflectors within tissue and the interfaces between tissues with different acoustic impedance. As a general principle, tissues that are acoustically homogeneous will generate low intensity signals, while structures with high gradients of acoustic impedance, such as bone or necrotic tissue, generate strong echoes and can obscure other structures deeper in the imaging plane. In a normal human brain, anatomical structures that give a hyperechoic signal on ultrasound imaging include the sulci, falx cerebri, choroid plexus, and vessel walls. In contrast, the ventricles and other spaces filled with cerebrospinal fluid are generally acoustically homogenous and create a low intensity hypoechoic signal. Lesions in the brain can have varying appearance depending on the mass density, necrotic infiltration, or fluid filled cavities but generally appear hyperechoic with areas of mixed echogenicity depending on the above specific features.

Intra-operative ultrasound, in the context of brain shift, was first introduced in 1997 by Bucholz (5) where they provided the first documented quantitative measurement of brain shift during hematoma and tumor neurosurgery. Before this, ultrasound had been previously introduced as an intra-operative neurosurgical tool to assist in small lesion identification in the context of arterio-venous malformation surgery by Chandler in 1987 (6). Since these initial publications, numerous investigators have implemented unique applications and procedures to harness this low-cost and widely available intra-operative imaging tool to gather real-time anatomical information for measuring and compensating for brain shift. The primary link between intra-operative imaging, such as ultrasound, and brain shift measurement or compensation is a registration procedure that relates intra-operative and pre-operative images to each other. In the context of iUS, the main challenge stemming from these registration procedures relates the widely different nature and quality of the iUS images as compared with the pre-operative MRI images. While voxel intensity of both modalities is directly dependent on the specific tissues imaged, there is an additional dependence for iUS on probe orientation and depth that leads to significant image intensity non-uniformity due to the presence of acoustic impedance transitions. The quality of individual ultrasound images is known to vary among users adding another obstacle when developing tools and methods to use this modality reliably for brain shift related interventions.

## Methods

This review followed the Preferred Reporting Items for Systematic Reviews and Meta-Analyses (PRISMA) 2009 guidelines without prior publication of the review protocol (7) and was performed using PubMed^1^ on August 17, 2020, to identify articles since 2015 with the specific words and phrases:

(“brain shift” OR brainshift) AND “ultrasound”

**Figure 2:**
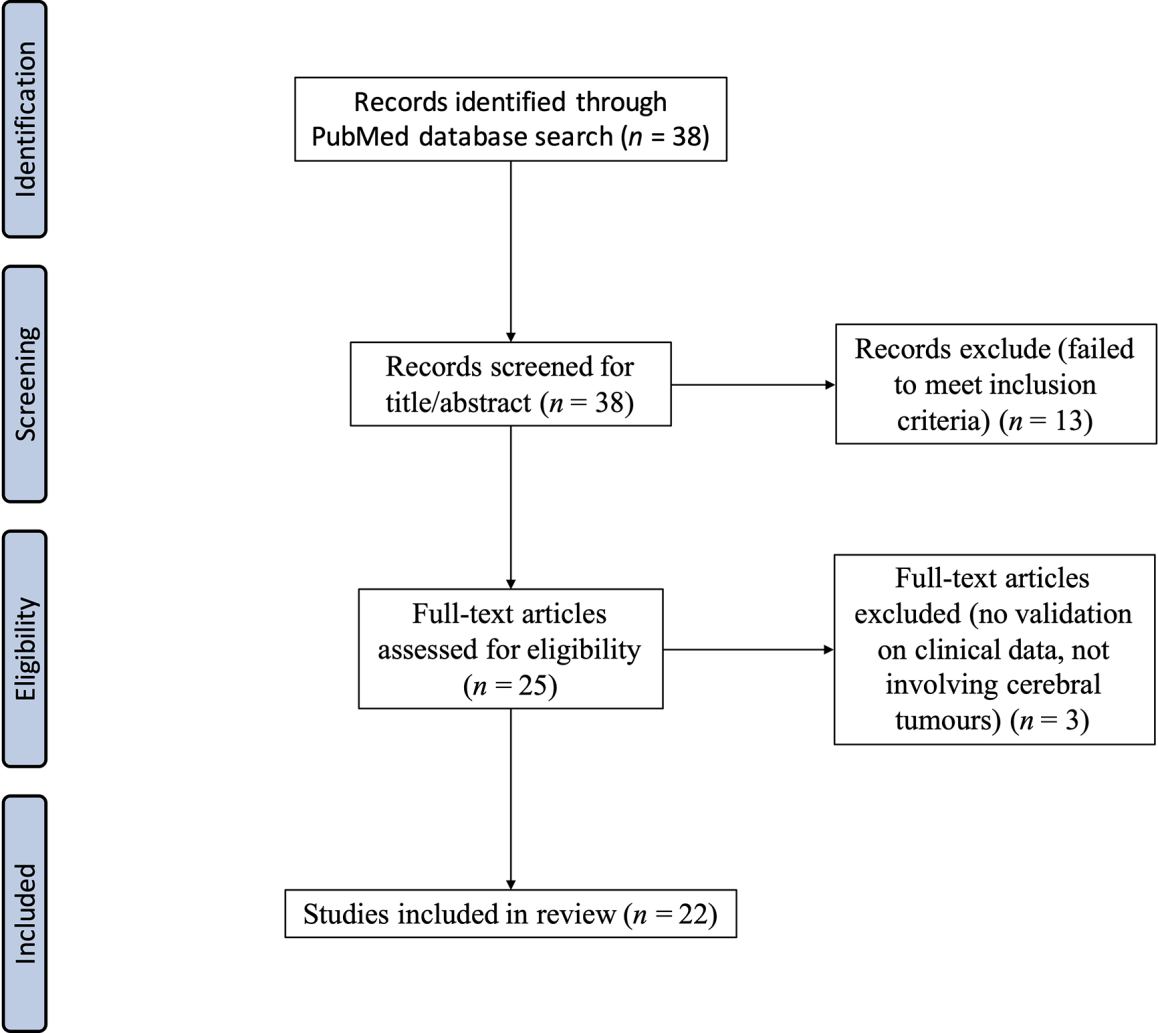
PRISMA diagram high-lighting the search strategy for reviewed articles.

The returned titles were screened for any non-English, duplicate, or clearly irrelevant entries, which were excluded. The inclusion criterion used during the selection was that the work must be focused on brain shift in the paradigm of image-guided neurosurgery of brain tumors. Exclusion criteria included review papers and work with animal-based studies and no clinical validation. For publications that were more mathematical in nature focusing on modeling, compensation, or prediction, validation of the methods on clinical datasets was required. Thirty-eight (38) relevant publications were found using the search query, of which 22 were included in this review. A PRISMA diagram is presented in **Figure 2**.

## Results

A summary of the papers reviewed, as they relate to the described taxonomy, location of the measured brain shift, pre-resection *vs.* post-resection measurement, and quantitative findings can be found in **Table 1**. In total, the list includes four qualitative retrospective case reviews, eight brain shift compensation methods papers, and the remaining 10 articles focused on prospective evaluation of brain shift measurement and/or compensation.

**Table 1:** Summary of the classification and quantitative results of reviewed articles (alphabetic order).

Reference	Classification	Measurement Locations	Mean Brain Shift (mm)	Compensation (mm)
Altieri et al. (8)	Qualitative Measurement	Retrospective review of cases	n/a	n/a
Canalini et al. (9)	Compensation (clinical)	Manual landmarks and segmented falx cerebri and sulci	Data from RESECT and BITE databases 3.49 ± 1.55 (RESECT, pre-resect) 3.54 ± 1.75 (RESECT, post-resect) 3.55 ± 2.28 (BITE)	1.56 ± 0.82 (RESECT, pre-resect, parametric) 1.36 ± 0.61 (RESECT, pre-resect, non-parametric) 2.29 ± 1.37 (RESECT, post-resect, parametric) 2.05 ± 1.12 (RESECT, post-resect, non-parametric) 2.98 ± 1.8 (BITE, parametric) 2.48 ± 2.67 (BITE, non-parametric)
Farnia et al. (10)	Compensation (phantom and clinical)	Sulci	Data from BITE database	2.14 ± 0.34 (BITE—13.71% improvement)
Farnia et al. (11)	Compensation (clinical)	Sulci, tumor boundary	Data from BITE database	1.87 ± 0.37 (BITE)
Farnia et al. (12)	Compensation (clinical)	Sulci, tumor boundary	Data from BITE database	1.83 ± 0.11 (BITE—15.37% improvement)
Frisken et al. (13)	Measurement and Compensation (clinical/modelling with thin plate splines [TPS] and finite element modelling [FEM])	Manual landmarks (ML) and automatic features (AF)	1.37 ± 0.81 (ML pre-resect) 2.79 ± 1.05 (ML mid-resect) 1.08 ± 0.65 (AF pre-resect) 2.31 ± 0.78 (AF mid-resect)	1.28 ± 0.63 (ML, TPS pre-resect) 1.82 ± 1.3 (ML, TPS mid-resect) 1.23 ± 0.68 (ML, FEM pre-resect) 1.37 ± 0.84 (ML, FEM mid-resect) 0.90 ± 0.62 (AF, FEM pre-resect) 1.05 ± 0.25 (AF, FEM mid-resect)
Frisken et al. (14)	Measurement and Compensation (clinical/modelling with TPS and FEM)	Manual landmarks (ML) and automatic features (AF)	5.3 ± 0.8 (ML, iUS_1_-MRI) 3.1 ± 2.2 (AF, iUS_1_-iUS_2_) 2.5 ± 1.8 (AF, iUS_1_-iUS_3_) 3.1 ± 1.7 (ML iUS_1_-iUS_2_) 2.5 ± 1.3 (ML iUS_1_-iUS_3_)	1.9 ± 0.6 (ML, iUS_1_–MRI) 2.2 ± 1.9 (AF, FEM, iUS_1_–iUS_2_) 1.8 ± 1.3 (AF, FEM, iUS_1_–iUS_3_) 3.6 ± 3.7 (ML, TPS, iUS_1_–iUS_2_) 2.4 ± 2.6 (ML, TPS, iUS_1_–iUS_3_)
Gerard et al. (15)	Measurement and Compensation (clinical)	ML and pixel misalignment (PM)	6.17 ± 2.21 (ML pre-resect) 5.62 (PM pre-resect)	2.43 ± 1.45 (ML pre-resect) 1.74 (PM pre-resect)
Ilunga et al. (16)	Compensation (algorithm validation)	Vascular segmentation	n/a	n/a
Iversen et al. (17)	Measurement and Compensation (clinical)	Manual landmarks (ML)	7.71 (mean, ML pre-resect) 5.12 (median, ML pre-resect)	4.47 (mean, ML pre-resect) 2.72 (median, ML pre-resect)
Liang et al. (18)	Qualitative Measurement and Compensation	Retrospective case review of gross total resection (GTR)	Image quality improved from poor/moderate to moderate/good	GTR in 22/26 cases using iUS–MRI fusion navigation vs. 6/19 using iUS without fusion
Machado et al. (19)	Measurement and Compensation (clinical)	ML for validation AF for registration	3.25 ± 1.93 Other data from BITE/RESECT	1.75 (AF, affine registration) 1.54 (AF, TPS registration) 1.85 (BITE, ML, affine) 1.52 (BITE, ML, TPS) 1.54 (RESECT, ML, affine) 1.49 (RESECT, ML, TPS)
Machado et al. (20)	Compensation (clinical)	Three database sets using ML	Data from BITE, RESECT, MIBS databases	2.28 ± 0.71 (BITE) 2.08 ± 0.37 (RESECT) 2.24 ± 0.78 (MIBS)
Masoumi et al. (21)	Compensation (clinical)	BITE and RESECT databases	Data from BITE and RESECT databases	2.77 ± 1.13 (RESECT) 2.82 ± 0.72 (BITE)
Morin et al. (22)	Compensation (modelling)	Vascular manual landmarks	2.63 ± 1.55	1.78 ± 1.42 (rigid registration) 1.83 ± 1.25 (constraint-based registration)
Petridis et al. (23)	Qualitative (clinical)	Retrospective review of iUS or no iUS for tumor resection	n/a	Target missed 0/15 cases (iUS) Target missed 5/19 cases (no iUS)
Prada et al. (24)	Compensation (clinical)	Anatomical and vascular landmarks	n/a	Reported as <2 mm in 42/58 cases and <3 mm in 58/58 cases after iUS–MRI registration
Riva et al. (25)	Measurement and Compensation (clinical)	Anatomic ML (sulci, gyri, ventricle, vessel)	5.9 ± 1.9 (pre-dura reflect) 6.2 ± 2.3 (post-dura reflect) 7.5 ± 2.1 (post-resect)	2.7 ± 1.0 [pre-dura reflect (rigid)] 4.2 ± 1.6 [post-dura reflect (rigid)] 6.7 ± 2.5 [post resect (rigid)]
Steno et al. (26)	Qualitative	Lenticulostriate arteries (LSA) visualization	n/a	n/a
Steno et al. (27)	Compensation (clinical)	Extent of resection (EOR)	n/a	86.79% EOR (mean, with iUS) 75.85% EOR (mean, no iUS)
Xiao et al. (28)	Measurement and Compensation (clinical)	Manual landmarks (ML)–tumor border, sulci, gyri	7.22 ± 3.35 (ML, pre-resect)	1.73 ± 0.62 (ML, pre-resect)
Zhou and Rivaz (29)	Compensation (algorithm validation)	Manual landmarks (ML)	Data from BITE database	1.5 ± 1.4 (ML, pre-resect, non-rigid symmetric registration [NSR])

RESECT, REtroSpective Evaluation of Cerebral Tumors database (30); BITE, Brain Images of Tumors for Evaluation database (31); MIBS, Multimodal Images of Brain Shift database (19).

### Qualitative Retrospective Case Reviews

Since 2015, four groups have published qualitative analysis in the form of a retrospective case review of their center’s experience with using intra-operative ultrasound for neurosurgical guidance. The first was published in 2015 by Petridis et al. (23) that reviewed 34 patients undergoing low grade glioma (LGG) resection between 2011 and 2014 in a German center. The retrospective analysis compared iUS use for localization of surgical targets with cases where iUS was not performed. They found in the 15 cases where iUS was used that the surgical target was properly found for either resection or biopsy, whereas in five of 19 cases where iUS was not used, the target was missed. The improvement was qualitatively attributed to intra-operative update of real-time information about brain shift as provided by the iUS imaging during these cases.

In 2016, Steno et al. (26) described a qualitative use of iUS during resection of insular low grade gliomas (LGG) during awake resections but with a focus on visualization of the lenticulostriate arteries. These landmarks served to measure brain shift compensation and to guide increased extent of resection, when compared to non-iUS interventions, without creating any new deficit while being nearby anatomic brain structures with important functional roles. Overall, their retrospective review of six cases demonstrated this to be a useful tool for this anatomical location of LGG. In 2018 (27), this group published a follow-up cohort case series of 49 patients undergoing awake resections for insular LGG nearby eloquent cortical and subcortical structures with 21 cases using only neuronavigation and the remaining 28 using iUS guidance. The mean extent of resection was significantly improved with iUS guidance (87 *vs.* 76%) without the addition of any new functional neurologic deficit.

Altieri et al. (8) describe a retrospective analysis of 264 patients with high-grade gliomas undergoing resection with neuronavigation and iUS guidance at the University of Turin between 2013 and 2016. The goal of their work was to improve the detection and characterize the echogenicity—the visual characteristics on ultrasound—of both normal and pathologic anatomical structures using different probes. The main challenge identified by the analysis, as often reported, was related to the surgeon’s comfort in interpreting the anatomy in oblique planes, a characteristic that increased with iUS experience.

Finally, in 2019, Liang et al. (18) published a retrospective case review on a cohort of patients that underwent iUS alone without registration during resection and iUS with pre-operative MRI registration to review the extent of resection (EOR) improvement. Of the 45 total patients reviewed, only 6/19 cases using iUS alone achieved gross total resection (GTR) whereas 22/26 (85%) cases using MRI registered to iUS had GTR. This significant clinical improvement was attributed primarily to the comfort and quality of using MRI images for guidance after registration as compared to iUS images alone. The authors also described a significantly lower postoperative morbidity rate in the iUS registration group and concluded that iUS–MRI registration is an essential tool to improve EOR and functional protection.

### Brain Shift Compensation Based on Clinical Datasets

Currently, there exists only two widely used and publicly available clinical databases with pre-operative MRI and iUS images that can be used for new brain shift compensation registration or predictive modeling algorithm validation: the Brain Images of Tumors for Evaluation (BITE) (31) and the REtroSpective Evaluation of Cerebral Tumors (RESECT) (30). Both databases have different internal limitations; however, they provide a necessary tool for comparison of brain shift compensation method development. Many authors (over 80 citations for BITE and over 30 for RESECT) have used these databases for the development and validation of innovative techniques. The quantitative results of the eight articles reviewed are in **Table 1**, and described below in more detail.

Farnia et al. have recently described brain shift compensation in a series of three articles (10–12) through matching of echogenic structures, specifically sulci, and optimization of the residual complexity value in the wavelet domain, a strategy to balance between feature and intensity-based registration approach advantages in multi-modal registration. With the introduction of the method in 2015, they validated the novel approach on both phantom and the BITE datasets, demonstrating a noted robustness to noise which is commonly encountered in iUS imaging. The following updates to their methods in 2016 and 2018 focused on improving computational time and the addition of a joint co-sparsity function to obtain a clinically acceptable and useful algorithm for intra-operative use. They report a registration accuracy of 0.90–1.82 mm depending on the method being evaluated. In all three of their works, they have shown significant improvement for both accuracy and efficiency that only lacks validation in a prospective setting.

Zhou and Rivaz 2016 (29) propose a non-rigid symmetric registration framework focused on pre- and post-resection ultrasound images to compensate for brain shift and assess for residual tumor that is difficult to assess on normal post-resection images due to the immense post-operative changes when compared with pre-operative MRI. This novel framework was validated on pre- and post-resection ultrasound images from the BITE database to identify “outlier regions” that may be consistent with possible residual tumor. The registration showed acceptable registration with reported accuracy, on the order of 1.5 mm, between the sets of images with the main drawback being long computation times not conducive to clinical workflow.

Continuing with the theme of novel registration strategies for brain shift compensation, in 2019, Masoumi et al. (21) describe an approach based on affine transformation that utilized a covariance matrix adaptation evolutionary strategy (CMA-ES) to optimize the registration. This work built upon their previous work in 2018 (32) that used a gradient descent optimization^2^. The method was evaluated on both the BITE and RESECT databases with statistically significant improvement of the mean target registration error (mTRE) on the order of 2.8 mm. Their proposed fully automatic registration improvement offers another option for iUS–MRI brain shift correction. The main advantages of this work compared to similar methods include an optimization step that is less susceptible to patch sizes and noise and is reported as the first use of CMA-ES specifically for MRI and US images.

In 2019, Canalini et al. (9) described a segmentation-based registration approach for brain shift compensation where the falx cerebri and different sulci were automatically segmented in pre-resection iUS volumes on the dura mater and used to register with iUS at different phases of the operation. The method is based on a trained convolutional neural network using manually annotated structures in the pre-resection ultrasound that are then used to segment and register the corresponding structures ad different phases of the operation. In contrast to previous work done, in this domain their solution focuses on iUS–iUS registration rather than iUS–MRI registration. They validated their method by comparing the mTRE between manually identified landmarks from the BITE and RESECT databases and showed significant improvement among both.

In one of the more complete series of brain shift compensation methodology papers, Machado et al. (19) published a registration procedure based on automatic feature detection followed by nearest-neighbor descriptor matching and probabilistic voting models similar to a Hough transform focused on scale-invariant features (SIFT). Their method was validated on two publicly available databases (BITE and RESECT) and, additionally, prospectively validated on a nine patient case series that they describe as the Multimodal Images of Brain Shift (MIBS) database. They report accuracy on the order of 2.2 mm with efficient registration results on all three data sets without the need to manually identify landmarks for evaluation. Within the same vein, in 2019 (20), this group described a correlation-based approach for brain shift compensation through extraction of multi-scale and multi-orientation attribute vectors with robust similarity measures on these attributes while simultaneously explicitly handling field-of-view differences between images as an approach to improve generalization and accuracy across different publicly available datasets. Their approach was validated on the BITE, RESECT, and MIBS databases, and tested against 15 other accepted multimodal registration algorithms. They consistently obtained one of the best results across the three datasets without deviation from their predefined parameters (compared to the often dataset specific tuning described in other papers). This approach highlights the potential need for more robust similarity functions and automatic feature detection frameworks that can be generalizable to the limited public data for future algorithm development.

### Prospective Brain Shift Measurement and Compensation

While retrospective data is important for method development and testing, it is critical to evaluate with prospective data to see how well methods generalize. The first of 10 prospective brain shift evaluation papers is from Prada et al. (24). They described their experience in 58 cases using an iUS-guided neuronavigation system. The measurements and compensation details for each individual case are not included in the article; however, they report that in 42 cases they were able to restore accuracy of the navigation system to below a critical threshold of 2 mm when compared with manually selected anatomic and vascular landmarks. In the 16 remaining cases, despite not reaching this critical clinical threshold, they were accurate to within 3 mm, and visualization of cerebral structures intra-operatively with iUS was achieved. Despite the lack of quantitative details on brain shift measurement and compensation this article highlights the expanding reach of iUS within the neurosurgical clinical community.

In 2017, Riva et al. (25) published an eight-patient case series to measure and compensate brain shift using 3D-iUS and an iterative deformation correction framework. Ultrasound was acquired at three time points during surgery: before dural opening, after dural opening, and following complete resection of the brain tumor. The goal of their work was to evaluate the robustness of mono-modal registration from serial iUS acquisitions at different time points in surgery in its ability to maintain accuracy of the navigation system and compensate for brain shift. The initial iUS volume is registered with a rigid transformation to the pre-operative MRI planning images using linear correlation of linear combination as a similarity metric. Following dural opening, iUS volumes are registered with the initial pre-dural iUS using both rigid normalized cross-correlation registration and deformable B-spline registration procedures and then applied to the original pre-operative planning volume. Their method was evaluated using expert neurosurgeon anatomic landmark identification to evaluate the target registration error. They report significant compensation of brain shift between the rigid registration of the initial iUS and pre-operative images both before (5.9 to 2.7 mm) and after (6.2 to 4.2 mm) dural removal with no significant improvement following complete resection (7.5 to 6.7 mm). The authors conclude that combining both mono- and multi-modal iUS registration in an iterative framework successfully measured and compensated brain shift and was easily integrated into the surgical workflow. This technique also has the potential to easily be expanded in other user-defined time points between those investigated in this work that could further help for more real-time brain shift correction throughout resection.

Xiao et al. (28) described a five patient case series evaluating a registration procedure between MRI tractography and iUS based on a correlation-ratio non-linear deformation framework. While the analysis was performed in a retrospective fashion and not intra-operatively, this article was included as the clinical data for which the algorithm was evaluated was not part of any previously published, publicly available database. This is the only report to describe MRI tractography–iUS registration in the context of brain shift measurement and compensation, and registration accuracy on the order of 1.7 mm was reported. As a relatively new imaging modality for surgical planning, tractography offers important information that, when accurately registered with pre-operative imaging, can help preserve white matter tracts important for proper brain function. The main limitation of this study results from the lack of data to validate their method and limited literature from which to draw for comparison. Despite this, they were successfully able to measure and compensate for brain shift in this short case series making it an intriguing avenue for future research.

In another prospective study, Gerard et al. (15) presented a unique approach to brain shift measurement and compensation with the combined use of iUS and augmented reality in a pilot study of eight cases using the Intraoperative Brain Imaging neuronavigation System (IBIS) (33). Brain shift was measured both with iUS and a compensation method based on gradient orientation alignment multimodal registration, as well as a calibrated augmented reality view where two-dimensional pixel misalignment error in a specified view was reported to provide both a qualitative and quantitative assessment of the associated brain shift. The main drawback in this work relates to the reporting of non-universal metric of pixel misalignment errors and the limited number and subjectivity of the manually identified landmarks. Despite these limitations, the authors demonstrate a combination of complementary technologies but require more extensive validation.

In 2019, Frisken et al. (13) describe a two-patient proof of concept study for brain shift measurement and compensation using thin plate spline registration and finite element method (FEM) modeling using physical and geometric constraints along with different material known biophysical properties of different tissues. During these two cases, they measured brain shift with both manually identified landmarks and automatic features using the SIFT method (19) with similar results for both the manual and automatically detected features. The brain shift was then compensated using two independent methods, thin-plate splines and FEM modeling, and the results were compared with one another. The main drawback, as stated by the authors, is that they were unable to compare the behavior of FEM and thin-plate splines for the automatically detected features since these features were used to train the splines and resulted in near-zero residuals; however, the FEM method had better results when compared with the thin-plate spine method for the manually identified landmarks, and given the similarity of brain shift measured between both the automatic features and manual landmarks, it is possible the FEM may have outperformed on these features as well if the splines had been trained on different features. This preliminary work motivated a more complete study which was published in 2020 (14) using a similar methodology with additional measurement and compensation of brain shift with serial iUS (*i.e.*, ultrasounds at multiple time points during the operations) and registration in a series of 19 cases. In their follow-up prospective study, the authors conclude that the FEM method provided more consistent brain shift correction and better compensation at locations further from the driving feature displacement than the thin-plate splines; however, in the cases with smaller deformations, the thin-plate splines performed better but without statistical significance. These results highlight the fact that multiple strategies are likely to be required when trying to account for brain shift in real-time and may evolve even throughout a single procedure.

### Prospective Brain Shift Measurement and Compensation Using Cerebral Vasculature

Contrast-enhanced ultrasound (CEUS) is a technique not often used in neurosurgical procedures; however, in 2016, Ilunga-Mbuyamba et al. (16) report on using CEUS for vascular structure identification and brain shift compensation in a series of 10 patients. The difficulty in reviewing this work for this article stems from the fact that only the similarity measures between the pre-segmented MRI images and the CEUS segmentation after registration are reported with no absolute registration error. Looking past this limitation, nine of the 10 cases evaluated in this report had successful brain shift compensation—reported as usable for clinical guidance—suggesting this unique approach could provide useful in highly vascular regions of operation.

Morin et al. (22) also focus on cerebral vasculature in a constraint-based biomechanical simulation of brain shift compensation for a series of five patients undergoing neurosurgery with iUS guidance. Each patient underwent a patient-specific biomechanical model built from pre-operative imaging which is intra-operatively registered with both iUS B-mode and Doppler imaging after a constraint-based simulation of the shift of the cerebral vascular tree. Manually chosen landmarks are used to assess the total brain shift and validate the compensation with reported accuracy on the order of 1.8 mm. The authors compared their work to their previously described rigid registration methods/techniques with successful results and having a workflow that is efficient for clinical integration.

In another prospective study, Iversen et al. (17) describe their experience using the CustusX platform (34) in a series of 13 patients. Intra-operative ultrasound was acquired pre-resection to update the guidance system in all 13 cases, and the amount of brain shift and subsequent compensation following registration with pre-operative MRI was evaluated using manual placed anatomic landmarks. They report that their system was deemed accurate enough for tumor resection guidance in nine of 13 cases following neurosurgeon evaluation and showed significant brain shift compensation in all 13 of their cases. The mean reported registration error was on the order of 4 mm with the median being 2.7 mm. This work highlights experience with one of the few open-source neuronavigation systems that support intra-operative ultrasound acquisition prospectively during navigation.

## Discussion and Conclusion

Brain shift is a very complex problem that has many pre- and intra-operative contributing factors. Strategies for measuring and compensating for brain shift continue to evolve, and intra-operative ultrasound continues to show promise as an effective, efficient, and low-cost solution for intra-operative accuracy management. Indeed, the rate of publication of intra-operative ultrasound brain shift related work has seen an increase from two to three articles per year, from 2005 to 2015, to eight to 10 articles per year since 2015. One of the primary issues with the current research landscape is that mathematical tool development in the form of registration, FEM, and predictive modeling continues to progress at a fast rate, but validation is repeatedly performed on a small cohort of publicly available retrospective data, that, while invaluable to the field, is nearly a decade old and may not accurately portray the quality and character of imaging used for guidance and surgical planning today. Additionally, as highlighted in Macahado et al. (19), many of these publicly available datasets require cohort-specific parameter tuning, and the compensation methods presented do not generalize well over the entirety of available data.

Indeed, one of the major needs for the field is newer and larger publicly available clinical datasets, such as that in the Brain Images of Tumors for Evaluation (BITE) (31), Retrospective Evaluation of Cerebral Tumors (ResECT) (30) databases. The Multimodal Images of Brain Shift (MIBS) (19) dataset is interesting, but not publicly available. Currently there are roughly 50 cases of pre-operative and intra-operative data freely available for research, a small number that reduces the strength and quality of validation and generalization of these compensation procedures. Another database that has yet to be published publicly but that has been described is the Brain Images of Tumors for Evaluation 2 (BITE2) database (35) which aims to build on the strengths of the original BITE database from the same group. This form of data sharing will be instrumental in more reliable and appropriate validation of these methods that reflect the modern pre- and intra-operative imaging landscape in neurosurgical oncologic procedures.

Among the many advances in the field since our last published review, the variety of applications that iUS has seen in neurosurgery over the last half-decade speaks to the extent to which the potential of the technology is being realized. Applications in cerebral vasculature, both as a tool for measuring brain shift, a feature for brain shift compensation, and a landmark for improving extent of resection are exciting for the field in terms of the broadness of how this tool will be used to treat patients and maintain accuracy for clinicians. With the advancement in technology comes additional challenges; as highlighted in the articles above there are numerous metrics reported for evaluation of brain shift compensation ranging from fiducial registration errors, target registration errors, extent of resection, segmentation similarity metrics, qualitative evaluations, and pixel misalignment errors. The difference in evaluation metrics and limited number of cases that techniques are evaluated makes comparison of new methodology especially difficult. In the context of extent of resection, for example, it is hard to know if the percentage increase in improvement is clinically significant and difficult to characterize and is attributed completely to brain shift compensation as opposed to specific patient and tumor anatomy.

Two additional points can be made with respect to reporting of results. While a standard for reporting target registration accuracy is desirable and some of the methods described here report accuracies better than 2mm, are these results useful clinically? Resection metrics, such as EOR and GTR provide valuable clinical information but given their value being relative in nature, it is difficult to compare them with other objective measures not specifically related to the tumor volumes. For example, Steno 2018 (27) reports a small but statistically significant improvement in EOR (from 76 to 87%), while Liang 2019 (18) reports an increase of tumor GTR from 32% without ultrasound navigation to 85% with ultrasound-corrected navigation. Both studies demonstrate statistically significant objective and clinical benefits of using iUS-based technology but are near impossible to compare with work that uses registration errors to quantify their results. The lack of a universally accepted evaluation metric and the non-reporting of absolute registration errors when assessing brain shift compensation remain a major challenge in the field for which there is no clear solution currently.

Intra-operative ultrasound for surgical guidance is a well-established tool and has seen applications in many organ systems including: hepatobiliary, genitourinary, lung, mediastinum, vascular, and breast (36). In many of the above applications, US has evolved from a complementary tool to one that has become almost standard-of-care for therapeutic intervention, especially within the hepatobiliary system. In vascular surgery, both within the cardiac and peripheral systems, both B-mode and Doppler iUS have been used, often to assist with surgical repair and to assess the adequacy of the repaired tissue (36, 37). It also plays a very important role in the vascular reconstruction phase of transplantation surgery for flow assessment and minimizing vascular complications (37). Additionally, there has been significant work using iUS navigation in the context of skull-based (38, 39). One of the main challenges during skull base tumor surgery is identifying the relationships between the lesion and principal intracranial vessels which are often mediated by neuronavigation systems. While inaccuracies due to brain shift at the skull base are generally minimal, there can still be other sources of inaccuracy making the pre-operative navigation images less reliable (39). Intra-operative US, often in the form of Doppler imaging and contrast-enhanced B-mode, can help improve the understanding of the skull-base and intracranial vessel relationship to avoid vascular damage and assist with lesion resection (38). The application of this technology in the brain adds an additional level of complexity as it is primarily used as a tool for re-calibrating MRI images for guidance as opposed to direct guidance. Despite this, it is important to learn from both the successes and failures from the many years of experience in these other surgical domains to maximize the potential of this technology and to avoid repeat failures or strategies that have proven to be inefficient from both clinical and technical points of view.

A final point of discussion stems from the timing of brain shift correction. While accurate navigation seems to be imperative for pre- and early perioperative planning, it is unclear on both how often and at which specific time points during surgery accurate brain shift correction correlated with pre-operative MRI is necessary. Once a neurosurgeon has begun operating and has an open cavity from which they can then see the surrounding anatomy comfortably, are highly accurate navigation images imperative to improve the success measure of that operation? As we saw reported in this review, Steno et al. (26, 27). evaluated the improvement of EOR with frequently updated navigation images for resections; however, very few studies report on these types of clinical outcomes, and there currently does not exist any report evaluating both perceived need for improved navigation images from surgeons or the objective analysis on the effects of the workflow and large-scale clinical outcomes. As iUS technology becomes more reliable and easily accessible, it will be important to have studies that identify optimal times of surgery where navigation accuracy is of high importance to improve clinical outcomes while not disrupting surgical workflow. To push the discussion to a further extreme, one may ask if we need to update navigation at all. With real-time imaging, like that provided through iUS, showing up-to-date anatomy and even functional information when Doppler mode is used, perhaps a better strategy would be to focus on improving surgeon comfort and technical proficiency with iUS image interpretation to remove the need for correlation with pre-operative MRI images which seems to have an upper limit of accuracy. In some select cases; however, where resection and anatomy are complex and iUS images difficult to interpret, it may serve beneficial to combine the information with pre-operative MRI as is the practice now for a more complete integration of information.

It is clear from the increasing rate of publication, specifically in qualitative retrospective case reviews and quasi-quantitative analysis from different neurosurgery centers across the world that the comfort and training in using iUS during surgery is expanding and its potential being realized by more clinicians. Unfortunately, the lack of prospective evidence continues to limit the overall reliability of the technology. Moving forward it will be imperative for multi-center prospective trials that focus on improving clinical criteria among patients undergoing iUS surgical guidance for tumor resections for this technology to make the next step and broaden into more clinical practices worldwide. With continued improvement on ultrasound hardware including portable probes with a smaller footprint such as the Clarius, Lumify, and Butterfly IQ, further support and easier clinical workflow integration for future trials is possible.

In conclusion, the growth of iUS in the field of neurosurgery is exciting and encouraging for both clinicians and researchers and continues to show major promise as a multi-faceted tool for measuring and compensating brain shift and improving both the safety and completeness of neurosurgical tumor resections.

## Author Contributions

IG—literature review, data analysis, manuscript writing. MK-O—Figure development, data analysis, manuscript editing and writing. JH—Expert neurosurgical critique and manuscript review/writing. DS—Expert neurosurgical critique and manuscript review/writing. DC—Supervisor, expert neurosurgical critique and manuscript review/writing, data analysis verification. All authors contributed to the article and approved the submitted version.

## Funding

The following grants supported this research: Canadian Institute of Health Research (MOP-97820) and Natural Science and Engineering Research Council of Canada (CHRP 385864).

## Conflict of Interest

The authors declare that the research was conducted in the absence of any commercial or financial relationships that could be construed as a potential conflict of interest.

## Footnotes

1 http://www.ncbi.nlm.nih.gov/pubmed

2 Masoumi et al. (32) was published as an extended abstract at a conference and thus, as a full article, did not meet inclusion criteria for full review.
